# Differential Effects of Polyphenols on Insulin Proteolysis by the Insulin-Degrading Enzyme

**DOI:** 10.3390/antiox10091342

**Published:** 2021-08-25

**Authors:** Qiuchen Zheng, Micheal T. Kebede, Bethany Lee, Claire A. Krasinski, Saadman Islam, Liliana A. Wurfl, Merc M. Kemeh, Valerie A. Ivancic, Charles E. Jakobsche, Donald E. Spratt, Noel D. Lazo

**Affiliations:** Gustaf H. Carlson School of Chemistry and Biochemistry, Clark University, Worcester, MA 01610, USA; qzheng@clarku.edu (Q.Z.); mkebede@clarku.edu (M.T.K.); blee@clarku.edu (B.L.); ckrasinski@clarku.edu (C.A.K.); sislam@clarku.edu (S.I.); lwurfl@clarku.edu (L.A.W.); mkemeh@clarku.edu (M.M.K.); vivancic@clarku.edu (V.A.I.); cjakobsche@clarku.edu (C.E.J.); dspratt@clarku.edu (D.E.S.)

**Keywords:** polyphenols, resveratrol, epigallocatechin-3-gallate, insulin-degrading enzyme

## Abstract

The insulin-degrading enzyme (IDE) possesses a strong ability to degrade insulin and Aβ42 that has been linked to the neurodegeneration in Alzheimer’s disease (AD). Given this, an attractive IDE-centric strategy for the development of therapeutics for AD is to boost IDE’s activity for the clearance of Aβ42 without offsetting insulin proteostasis. Recently, we showed that resveratrol enhances IDE’s activity toward Aβ42. In this work, we used a combination of chromatographic and spectroscopic techniques to investigate the effects of resveratrol on IDE’s activity toward insulin. For comparison, we also studied epigallocatechin-3-gallate (EGCG). Our results show that the two polyphenols affect the IDE-dependent degradation of insulin in different ways: EGCG inhibits IDE while resveratrol has no effect. These findings suggest that polyphenols provide a path for developing therapeutic strategies that can selectively target IDE substrate specificity.

## 1. Introduction

**Figure 1:**
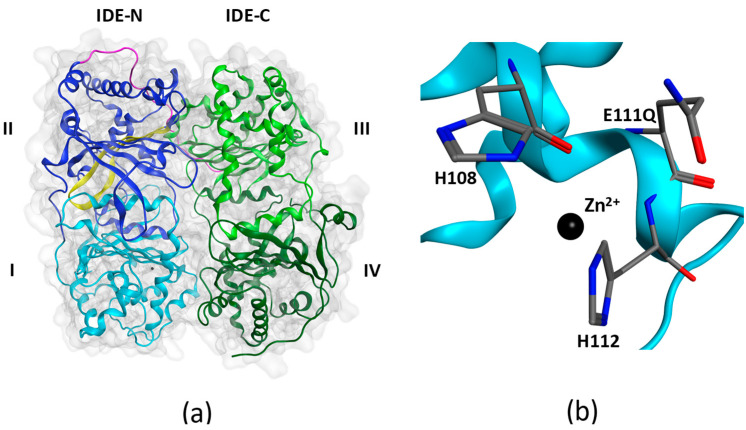
X-ray structure of insulin-degrading enzyme in its closed conformational state (PDB: 4PES). (**a**) IDE is composed of an N-terminal half (IDE-N) containing domains I and II, and a C-terminal half (IDE-C) containing domains III and IV. The two halves are joined by an unstructured linker (magenta). Domain I contains the HXXEH zinc-binding and catalytic motif. Domain II contains the exosite (yellow) in an anti-parallel β-sheet conformation. (**b**) The HXXEH motif includes H108 and H112 that coordinate Zn^2+^ and the catalytically important glutamate (E111) residue. When E111 is mutated to a glutamine as in 4PES, the enzyme becomes inactive.

Insulin-degrading enzyme (IDE), aka insulysin, is a ubiquitous zinc-dependent protease belonging to the M16 family of metalloendopeptidases [1]. Members of this family form a catalytic chamber, also referred to as crypt, the volume of which in IDE (~15,700 Å^3^ [2]) limits the length of substrates to less than 70 amino acids [3]. Biophysical studies using X-ray crystallography [2,4,5] and cryogenic electron microscopy [6] have revealed mechanisms by which IDE encloses and degrades a diverse group of metabolically important and pathologically relevant substrates including insulin, a key hormone for glucose metabolism, amyloid-β(1-42) (Aβ42), which self-assembles to form the proximate neurotoxic assemblies in Alzheimer’s disease (AD) [7], and islet amyloid polypeptide (IAPP), which self-assembles to form pancreatic amyloid in type 2 diabetes (T2D) [8]. IDE consists of two bowl-shaped halves, IDE-N and IDE-C, held together by an unstructured linker (Figure 1a). The flexibility of the linker allows IDE to exist in two major conformational states during its catalytic cycle: an open conformational state, which facilitates substrate entry and anchoring to IDE’s exosite; and a closed conformational state, which expedites substrate degradation. IDE bears the HXXEH catalytic motif arranged so that the two histidine residues (His108 and His112) coordinate Zn^2+^ and the catalytic role of Glu111 in the hydrolysis of peptide bonds is facilitated (Figure 1b).

**Figure 2:**
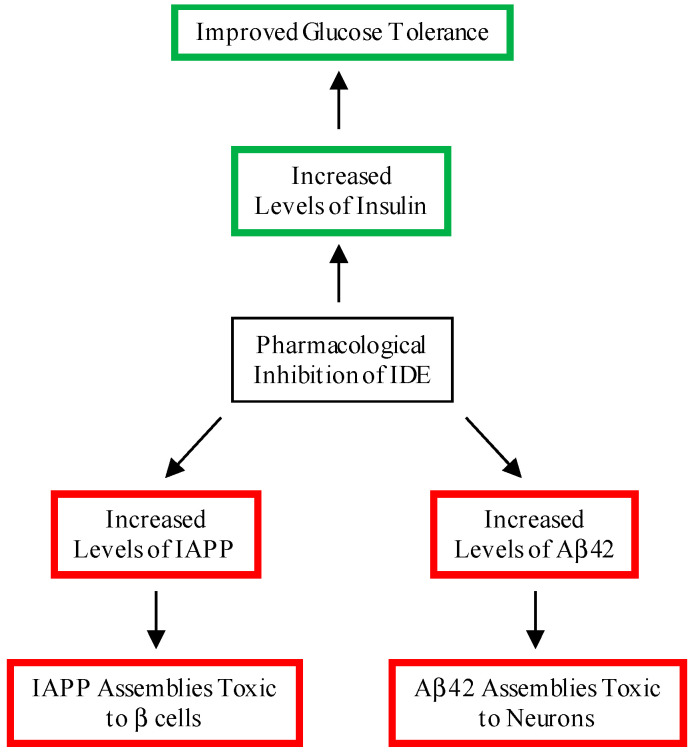
Beneficial and deleterious consequences of the pharmacological inhibition of IDE. A beneficial consequence of the inhibition of IDE is increased levels of insulin, leading to an improvement in glucose tolerance. Deleterious consequences include increased levels of Aβ42 and IAPP, leading to the formation of cytotoxic assemblies in the brain and pancreas, respectively.

Given IDE’s ability to degrade insulin, IAPP and Aβ42, it is no surprise that there has been strong academic and pharmaceutical interest in developing small molecules that modulate IDE’s activity [9,10,11,12]. Several small-molecule and peptidic IDE inhibitors have been developed and investigated for their effects on insulin levels in cells or mice. Ii1 [13] and P12-3A [14] inhibited degradation of extracellular insulin in cells and fibroblasts, respectively. BDM44768 [15], B35 [16], and 6bk [17] elevated plasma insulin levels in mice. Despite these advances, significant challenges remain, as noted in a recent review by Leissring et al. [12]. First and foremost, inhibiting IDE may lead to increased levels of IAPP and Aβ, increasing the risk for T2D and AD, respectively (Figure 2).

**Figure 3:**
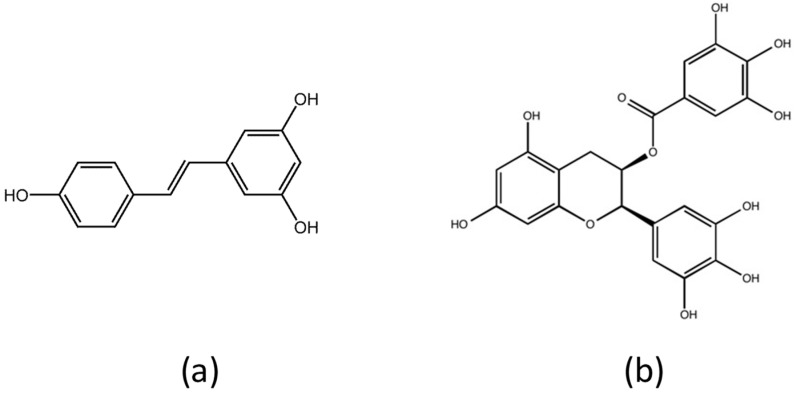
Chemical structures of polyphenols used in this study. (**a**) Resveratrol (C_14_H_12_O_3_) is found in red wine and red grapes. (**b**) Epigallocatechin-3-gallate (C_22_H_18_O_11_) is found in green tea.

An attractive alternative to the development of small-molecule inhibitors of IDE is a nutritional strategy that implicates polyphenols. Polyphenols are naturally occurring compounds that possess antioxidant [18,19,20], anti-inflammatory [19,20,21], and anti-amyloidogenic [22] properties. As such, polyphenols have been hypothesized to prevent T2D [23] and AD [24]. Recently, we showed that IDE’s activity toward Aβ42 is sustained in the presence of resveratrol (Figure 3a) [25]. The effect of resveratrol on the IDE-dependent degradation of insulin, the enzyme’s most physiologically important substrate, however, remains unknown. In this work, we used a combination of chromatographic and spectroscopy-based kinetic analysis to investigate the effects of resveratrol on IDE’s activity toward insulin. For comparison, similar experiments were conducted in the presence of epigallocatechin-3-gallate (EGCG, Figure 3b), which is approximately two times larger than resveratrol, and is similar in size to BDM44768 [15]. Our results show that EGCG inhibits the IDE-dependent degradation of insulin, whereas resveratrol has no effect, presumably because of its smaller size. The implications of these results in the development of IDE-centric therapeutic strategies for T2D and AD are discussed.

## 2. Materials and Methods

### 2.1. Insulin-Degrading Enzyme Expression and Purification

The bacterial expression vector encoding IDE fused to GST was kindly provided by Dr. Malcolm A. Leissring of the University of California at Irvine. GST-IDE was overexpressed in *E. coli* BL21-CodonPlus RIL cells (EMD Biosciences Inc., San Diego, CA, USA), and purified as previously described [25,26,27]. Cleavage of the GST tag was accomplished using GST PreScission protease and further purification of IDE was accomplished by standard gel filtration [25,26,27]. UV absorbance at 280 nm was used to determine the concentration of IDE using an extinction coefficient of ε_280nm_ = 113,570 M^−1^ cm^−1^ [28].

### 2.2. Preparation of Stock Solutions

Recombinant human insulin (99% pure), EGCG (>98% pure), and resveratrol (>99% pure) were purchased from Sigma-Aldrich (St. Louis, MO, USA). Stock solutions of insulin and EGCG were prepared in 50 mM Tris buffer (pH 7.4). Stock solutions of resveratrol were prepared in 100% ethanol. The concentrations of the stock solutions were detected by UV absorbance at the following wavelengths: 276 nm for insulin (ε_276nm_ = 6190 M^−1^ cm^−1^) [29]; 275 nm for EGCG (ε_275nm_ = 11,500 M^−1^ cm^−1^) [30]; and 306 nm for resveratrol (ε_306nm_ = 15,400 M^−1^ cm^−1^) [31]. All stock solutions were freshly prepared, and used immediately after preparation.

### 2.3. Circular Dichroism Spectroscopy

#### 2.3.1. Monitoring the Loss of Insulin’s Helical Circular Dichroic Signals with Digestion Time

We used circular dichroism spectroscopy to detect the changes in insulin’s secondary structure as its proteolysis proceeds. All far UV circular dichroic (CD) spectra were recorded at 37 °C using a JASCO J-815 spectropolarimeter equipped with a Peltier temperature control unit. Each sample was prepared with a volume of 200 μL. The substrate-to-enzyme molar ratio of insulin-to-IDE was 100:1 (20 μM insulin: 0.2 μM IDE) for limited proteolysis [26], and the molar ratio of insulin-to-polyphenol was set as 1:2 (20 μM insulin: 40 μM EGCG or resveratrol), similar to the molar ratio of Aβ42-to-resveratrol we previously used [25]. We determined that by setting the insulin concentration at 20 μM, CD spectra of good signal-to-noise ratios were recorded in the far UV range (i.e., from 260 to 198 nm). All samples were incubated in quartz cuvettes with a path length of 1 mm. Each CD spectrum was recorded from 260 to 198 nm using an averaging time of 1 s and four accumulations. All samples were kept at 37 °C in between recording of spectra. The Savitzsky–Golay method with convolution width equal to 7 was applied to smoothen all CD spectra.

#### 2.3.2. Kinetic Parameters from Insulin’s Helical Circular Dichroic Ellipticity at 222 nm

Steady-state kinetic parameters for the IDE-dependent degradation of insulin were determined using insulin’s helical circular dichroic ellipticity at 222 nm ([*θ*_obs(222nm)_]), as described in detail elsewhere [26]. Briefly, seven substrate solutions with concentrations of 15, 20, 25, 30, 50, 80, and 110 µM were prepared in 50 mM Tris buffer (pH 7.4). EGCG or resveratrol was added at a concentration of 20 or 40 μM. Proteolysis was initiated with the addition of IDE at a concentration of 1 µM. After mixing, the solution was transferred into a 1-mm path length quartz cuvette and loaded into the sample holder of our circular dichroism spectrometer that was pre-warmed to 37 °C. After a brief equilibration period, [*θ*_obs(222nm)_] was recorded for 5 min. The real-time [*θ*_obs(222nm)_] data were then used to calculate the amount of digested insulin ([DI]) using the equation below:

(1) $${\left[\mathrm{DI}\right]}_{t}={\left[I\right]}_{0}\times \left(1-\frac{{\left[\theta_{\mathrm{obs}\left(222\mathrm{nm}\right)}\right]}_{t}}{{\left[\theta_{\mathrm{obs}\left(222\mathrm{nm}\right)}\right]}_{0}}\right)$$

where [DI]*_t_* is the amount of digested insulin at real-time *t*; [I]_0_ is the initial amount of undigested insulin; and [*θ*_obs(222nm)_]*_t_* and [*θ*_obs(222nm)_]_0_ are the observed ellipticity at real-time *t* and observed initial ellipticity at time = 0, respectively. Plots of [DI] against time were used to determine the initial rates (*V*_0_) of insulin proteolysis by IDE. Michaelis–Menten (*V_0_* plotted against [*S*], Lineweaver–Burk (1/*V_0_* plotted against 1/[*S*]), and Hanes–Woolf ([*S*]/*V_0_* plotted against [*S*]) plots were then constructed from which the kinetic constants *K*_M_, *V*_max_, *k*_cat_ and *k*_cat_/*K*_M_ were determined.

### 2.4. Reversed Phase High Performance Liquid Chromatography

Proteolysis samples were prepared for RP HPLC analysis by setting the substrate-to-enzyme molar ratio to 100:1, and the polyphenol-to-insulin molar ratio to 2:1. These included: (1) insulin + EGCG + IDE (20 μM insulin + 40 μM EGCG + 0.2 μM IDE); and (2) insulin + RES + IDE (20 μM insulin + 40 μM RES + 0.2 μM IDE). Control samples that only contain insulin + IDE (20 μM insulin + 0.2 μM IDE) were also prepared. All reactions were started by the addition of IDE followed by incubation at 37 °C. Aliquots (200 μL) of each digests were removed at the desired time points and quenched by acidification to low pH with 15 μL of 5% (*v/v*) trifluoroacetic acid in water.

The IDE-dependent proteolysis of insulin was monitored using a Varian Pro Star 210 HPLC system, equipped with a ProStar 325 Variable Wavelength UV–Visible Detector. RP HPLC fractionation of insulin digests was carried out at room temperature using an analytical Agilent AdvanceBio mAb C4 column. Solvents A and B were 0.1% (*v/v*) formic acid in H_2_O and 0.1% (*v/v*) formic acid in acetonitrile, respectively. Aliquots (20 μL) of solutions containing insulin digests were injected into the HPLC manually and eluted with a 20-min linear gradient of 0–100% B at a flow rate of 1 mL/min. The elution of analytes was monitored by UV absorbance at 214 and 254 nm.

## 3. Results and Discussion

Insulin’s CD spectrum indicates a predominantly α-helical structure as indicated by the following features [32]: (1) a positive π → π* band at 195 nm, polarized ⊥ to the helix axis; (2) a negative π → π* band at 208 nm, polarized ‖to the helix axis; and (3) a negative n → π* band at 222 nm, also polarized ‖to the helix axis. Recently, we showed that the CD spectrum of insulin is sensitive to the extent of IDE-dependent degradation [26]. In particular, we noted that as proteolysis occurs, the intensities of insulin’s helical CD signals decrease with an increase in digestion time. The complete degradation of insulin by IDE is indicated by a CD spectrum with a single minimum near 198 nm, consistent with the dominant presence of unstructured or random coil peptides [26].

**Figure 4:**
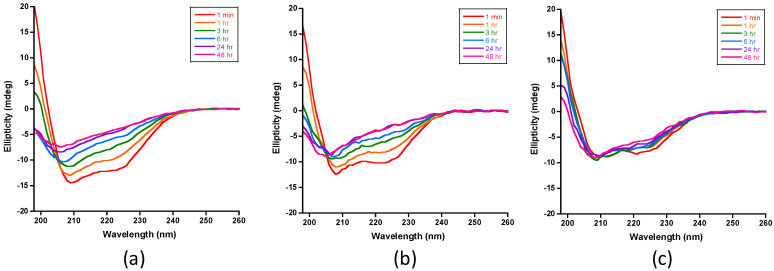
CD spectra of IDE-dependent degradation of insulin in the absence and presence of polyphenols at 37 °C. (**a**) Spectra recorded for insulin digests that do not contain polyphenols (control samples) show the progressive loss on insulin’s helical CD signals at 222, 208, and 198 nm, consistent with the loss of α-helical structure. (**b**) Spectra recorded for insulin digests in the presence of resveratrol were similar to the spectra recorded for the control samples, indicating that the polyphenol had no effect on IDE’s activity toward insulin. (**c**) Spectra recorded for insulin digests in the presence of EGCG showed that the helical signals of insulin persist, indicating that EGCG inhibits insulin proteolysis by IDE. The insulin concentration in all samples was set at 20 μM in 50 mM Tris buffer (pH 7.4). The substrate-to-enzyme molar ratio and the polyphenol-to-insulin molar ratio were set to 100:1 and 2:1, respectively. All samples were kept at 37 °C in between spectral acquisition.

Figure 4 presents the CD spectra of insulin IDE digests in the absence of polyphenols and in the presence of EGCG or resveratrol. The time-dependent spectra recorded in the absence of polyphenols (Figure 4a) were similar to those we reported recently [26]. The intensities of the signals at 222 and 208 nm decreased with time, indicating the progressive loss of α-helical insulin. Similar results were obtained in the digestions that contained resveratrol (Figure 4b), which suggest that the polyphenol has no effect on the IDE-dependent degradation of insulin. In sharp contrast, insulin’s CD helical signals persisted in the presence of EGCG (Figure 4c), indicating inhibition of the proteolysis of insulin by IDE. We noted, however, that the intensities of the helical signals, particularly at 222 and 198 nm, decreased, suggesting that the inhibition is partial and not complete.

**Figure 5:**
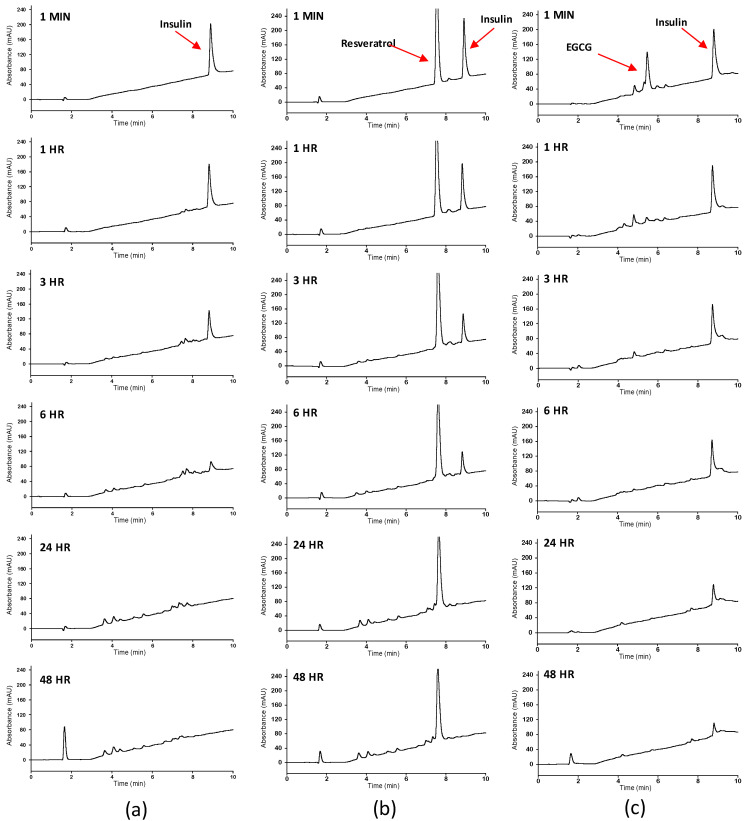
RP HPLC analysis of IDE-dependent degradation of insulin in the absence and presence of polyphenols at 37 °C. (**a**) Chromatograms of insulin digests in the absence of polyphenols (control samples) show the progressive loss of the insulin peak over the digestion period. This loss is accompanied by new peaks at shorter retention times due to the formation of insulin fragments. The chromatogram recorded for the 24-hr digest shows the absence of the insulin peak, indicating complete degradation of insulin. (**b**) Chromatograms of insulin digests in the presence of resveratrol are similar to the chromatograms in (**a**) with the exception of the peak for the polyphenol at a retention time of 7.5 min. Digestion of insulin is complete within 24 h. (**c**) Chromatograms of insulin digests in the presence of EGCG show that the digestion of insulin was not complete, even after 48 h of digestion. The intensity of the insulin peak, however, decreased with digestion time, suggesting that the inhibition by EGCG was partial and not complete. The intensity of the peak for EGCG decreased with incubation time presumably because of epimerization and/or dimerization.

We also used reversed phase HPLC to monitor the proteolytic activity of IDE toward insulin over the same incubation periods used in the digestions that yielded the CD spectra shown in Figure 4. Representative chromatograms of control samples are shown in Figure 5a. As the digestion time increased, the intensity of the insulin peak decreased and peaks at shorter retention times appeared due to the production of insulin fragments. Digestion is complete within 24 h. Figure 5b presents chromatograms of the digests in the presence of resveratrol. With the exception of the peak for resveratrol, the chromatograms were similar to those of the control samples. Insulin degradation was also complete within 24 h. Figure 5c shows the chromatograms of the digests in the presence of EGCG. In contrast to the corresponding chromatograms in Figure 5a,b, the chromatograms of the 24- and 48-h digests showed the presence of the insulin peak indicating inhibition of IDE-dependent degradation. The intensity of the insulin peak, however, decreased with time, suggesting that the inhibition of IDE was not complete. The intensity of the peak for EGCG decreased with incubation time, presumably because it epimerizes to gallocatechin-3-gallate and/or dimerizes to form digallate dimers [22,33]. Our cumulative CD (Figure 4) and RP HPLC (Figure 5) results clearly demonstrate that resveratrol has no effect on IDE’s activity toward insulin. EGCG, on the other hand, partially inhibits IDE.

**Scheme 1:**
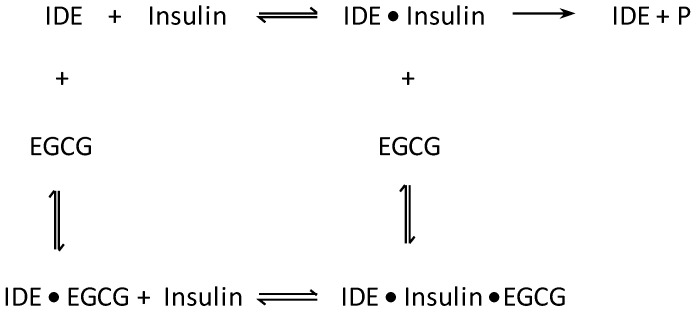
Schematic representation of the mixed inhibition of IDE-dependent degradation of insulin by EGCG. EGCG binds to either IDE or IDE●insulin complex, leading to a decrease in catalytic activity.

**Figure 6:**
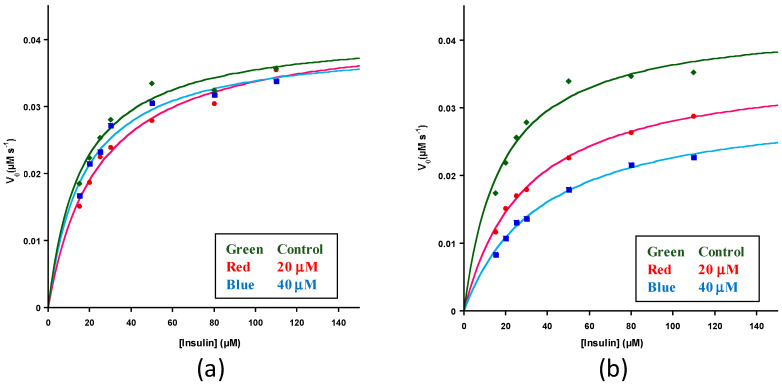
Kinetics of IDE-dependent degradation of insulin in the absence and presence of polyphenols at 37 °C. (**a**) Michaelis–Menten plots for insulin digests in the absence and presence of resveratrol were similar to one another, indicating that the polyphenol has no effect on IDE’s activity toward insulin. (**b**) Corresponding plots for the digestions in the absence and presence of EGCG clearly show that IDE’s activity decreased as the concentration of EGCG increased. Plots for polyphenol concentrations of 0, 20, and 40 μM are shown in green, red, and blue, respectively. Each data point represents the mean from three trials. The solid lines are fits to the Michaelis–Menten equation.

Next, the steady-state kinetic parameters for the IDE-dependent degradation of insulin in the absence and presence of polyphenols were determined using insulin’s helical CD ellipticity at 222 nm ([*θ*_obs(222nm)_]). Because our analysis was limited to 5 min of digestion time, complications due to the epimerization and oxidation of EGCG were circumvented. Figures S1a and S2a present representative real-time ellipticity data obtained from proteolysis experiments in the presence of resveratrol and EGCG, respectively. As digestion proceeded, [*θ*_obs(222nm)_] became less negative indicating loss of helicity. We calculated the amount of digested insulin [DI]*_t_* using Equation (1) and these were plotted against digestion time (Figures S1b and S2b). Linear regression analysis yielded *V*_0_, the initial velocity (or initial rate) of insulin proteolysis. Figure 6a,b present the resulting Michaelis–Menten plots for the digestions carried out in the presence of resveratrol and EGCG, respectively, at polyphenol concentrations of 0 (control), 20, and 40 μM. Lineweaver–Burk and Hanes–Woolf plots for the digestions in the presence of resveratrol and EGCG are shown in Figures S3 and S4, respectively. The Michaelis–Menten (Figure 6a), Lineweaver–Burk (Figure S3), and Hanes–Woolf (Figure S3) plots for resveratrol are invariant to concentration, indicating that the polyphenol has no effect on IDE’s activity. In sharp contrast, the corresponding plots for EGCG clearly indicate that IDE’s activity decreased as the concentration of the polyphenol increased (Figure 6b and Figure S4). Table 1 summarizes the kinetic constants obtained from the Michaelis–Menten plots. Similar values were obtained from the Lineweaver–Burk (Table S1) and Hanes–Woolf (Table S2) plots. IDE’s catalytic efficiency, as given by *k_cat_/K_M_*, was not affected by resveratrol. In sharp contrast, *k_cat_/K_M_* decreased by 54 and 67% relative to the control in the presence of 20 and 40 μM of EGCG, respectively. The Lineweaver–Burk plots (Figure S4a) show straight lines with different slopes and a common intercept that is not on the 1/*V*_0_ axis, suggesting mixed inhibition by EGCG. Mixed inhibitors bind at a site separate from the enzyme’s active site, but may bind to either the enzyme or enzyme-substrate complex [34]. A schematic representation of the mixed inhibition by IDE is shown in Scheme 1.

In vivo studies on animals have identified beneficial effects of EGCG including decreased adipose mass [35], reduction in body weight [36], and improvement in glucose homeostasis [35,36,37]. The effects of EGCG on glucose metabolism associated with T2D have been reported to be mediated in several ways including decreased gluconeogenesis [38], increased insulin sensitivity [39], and increased glucose uptake in skeletal muscle [40], which is an important regulator of glucose homeostasis [41]. The direct molecular targets of EGCG in vivo are not known. This work shows that EGCG directly targets IDE, and in doing so, the IDE-dependent degradation of insulin is inhibited, providing molecular basis for the improvement in glucose homeostasis observed in the in vivo studies.

How might our resveratrol results be used in the development of therapeutic and/or preventive strategies for AD? Recently, we showed that resveratrol sustains IDE activity toward Aβ42 monomer [25]. This conclusion, together with this work’s finding that resveratrol does not affect IDE’s activity toward insulin, has an important implication. Given that insulin and Aβ42 are IDE’s most physiologically important and most pathologically significant substrates in the brain, respectively, resveratrol is an IDE substrate-selective activator. Resveratrol’s selectivity toward Aβ42 and its ability to cross the blood–brain barrier [24,42,43] suggest that this polyphenol can potentially act as an ideal naturally-occurring molecule for an IDE-centric therapeutic for AD.

## Figures and Tables

**Table 1:** Steady-state kinetic parameters for the IDE-dependent proteolysis of insulin determined from Michaelis–Menten plots.

Polyphenol	*K*_M_ (M)	*k*_cat_ (s^−1^)	*k*_cat_/*K*_M_ (M^−1^S^−1^)
None	1.8 ± 0.2 × 10^−5^	0.043 ± 0.0006	2.4 ± 0.3 × 10^3^
None/Ethanol *	1.6 ± 0.2 × 10^−5^	0.041 ± 0.001	2.6 ± 0.3 × 10^3^
Resveratrol (20 μM)	2.6 ± 1.2 × 10^−5^	0.043 ± 0.003	1.8 ± 0.6 × 10^3^
Resveratrol (40 μM)	1.7 ± 0.3 × 10^−5^	0.040 ± 0.004	2.3 ± 0.1 × 10^3^
EGCG (20 μM)	3.2 ± 1.2 × 10^−5^	0.037 ± 0.002	1.3 ± 0.4 × 10^3^
EGCG (40 μM)	3.7 ± 0.9 × 10^−5^	0.031 ± 0.0008	0.83 ± 0.2 × 10^3^

* Control experiments to test the effect of ethanol that was used to dissolve resveratrol.

## Data Availability

Data is contained within the article.

## Supplementary Materials

The following are available online at https://www.mdpi.com/article/10.3390/antiox10091342/s1, Figure S1: Early-stage kinetics of IDE-dependent degradation of insulin in the absence and presence of resveratrol using insulin’s observed ellipticity at 222 nm [*θ*_obs(222nm)_], Figure S2: Early-stage kinetics of IDE-dependent degradation of insulin in the absence and presence of EGCG using observed ellipticity at 222 nm [*θ*_obs(222nm)_], Figure S3: Kinetics of IDE-dependent degradation of insulin in the absence and presence of resveratrol at 37 °C, Figure S4: Kinetics of IDE-dependent degradation of insulin in the absence and presence of EGCG at 37 °C, Table S1: Steady-state kinetic parameters of the IDE-dependent proteolysis of insulin determined from Lineweaver–Burk plots, Table S2: Steady-state kinetic parameters of IDE-dependent proteolysis of insulin determined from Hanes–Woolf plots.
