# Supplementary material for: Differential Effects of Polyphenols on Insulin Proteolysis by the Insulin-Degrading Enzyme
Source: Antioxidants (Basel). 2021 Aug 25;10(9):1342. doi: 10.3390/antiox10091342 (PMC8467823; doi:10.3390/antiox10091342)
Supplement: Supplementary file 1 [file antioxidants-10-01342-s001.zip › antioxidants-1335579-supplementary.pdf]

SUPPORTING INFORMATION

Differential Effects of Polyphenols on Insulin Proteolysis
by the Insulin-degrading Enzyme

Qiuchen Zheng,[‡] Micheal T. Kebede,[‡] Bethany Lee,[‡] Claire A. Krasinski, Saadman Islam, Liliana A. Wurfl, Merc M. Kemeh, Valerie A. Ivancic, Charles E. Jakobsche, Donald E. Spratt, and Noel D. Lazo*

Gustaf H. Carlson School of Chemistry and Biochemistry, Clark University, Worcester, MA 01610 USA

[‡] These authors contributed equally to this work.
*Correspondence: nlazo@clarku.edu

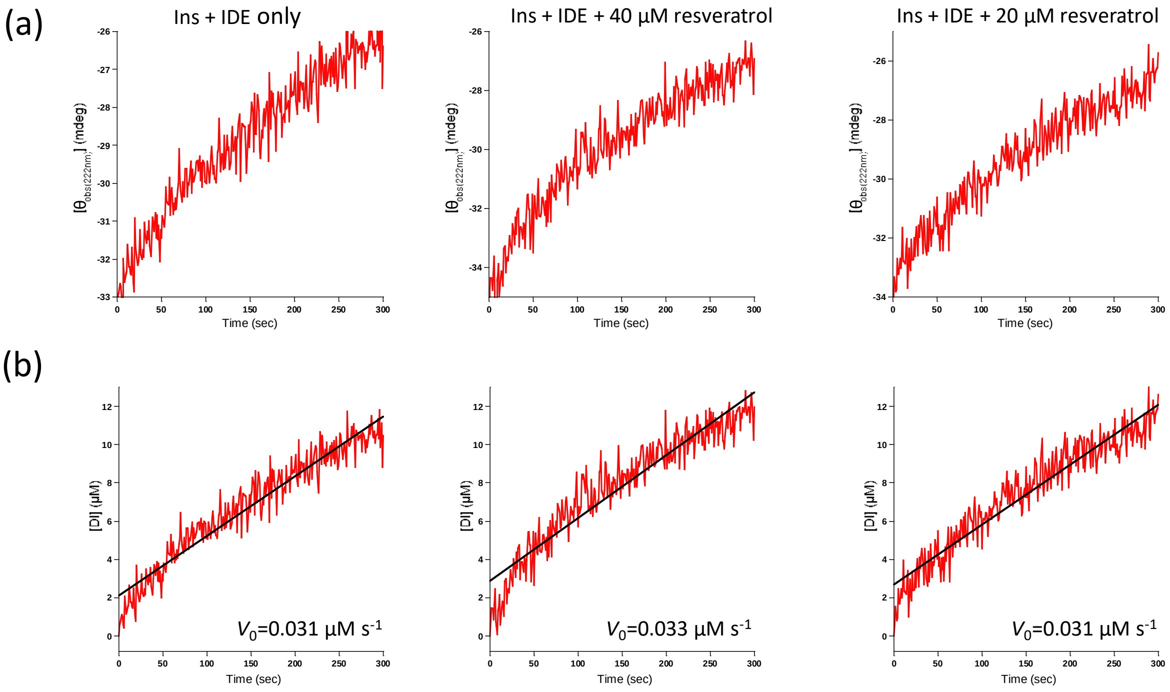

Figure S1. Early-stage kinetics of IDE-dependent degradation of insulin in the absence and presence of resveratrol using insulin's observed ellipticity at 222 nm [$\theta_{obs(222\ nm)}$]. (a) Representative real-time plots of [$\theta_{obs(222\ nm)}$] versus initial digestion time (0 - 300 s) in the absence and presence of 40 and 20 μM resveratrol at 37 °C. (b) Corresponding real-time plots of digested insulin [DI], calculated using Equation 1, versus digestion time. The R^2 values of the fitting of the data to straight lines were determined to be 0.92, 0.92, and 0.90 (left to right). V_0 was obtained from the slope of each line. The concentration of insulin in these samples was set at 50 μM.

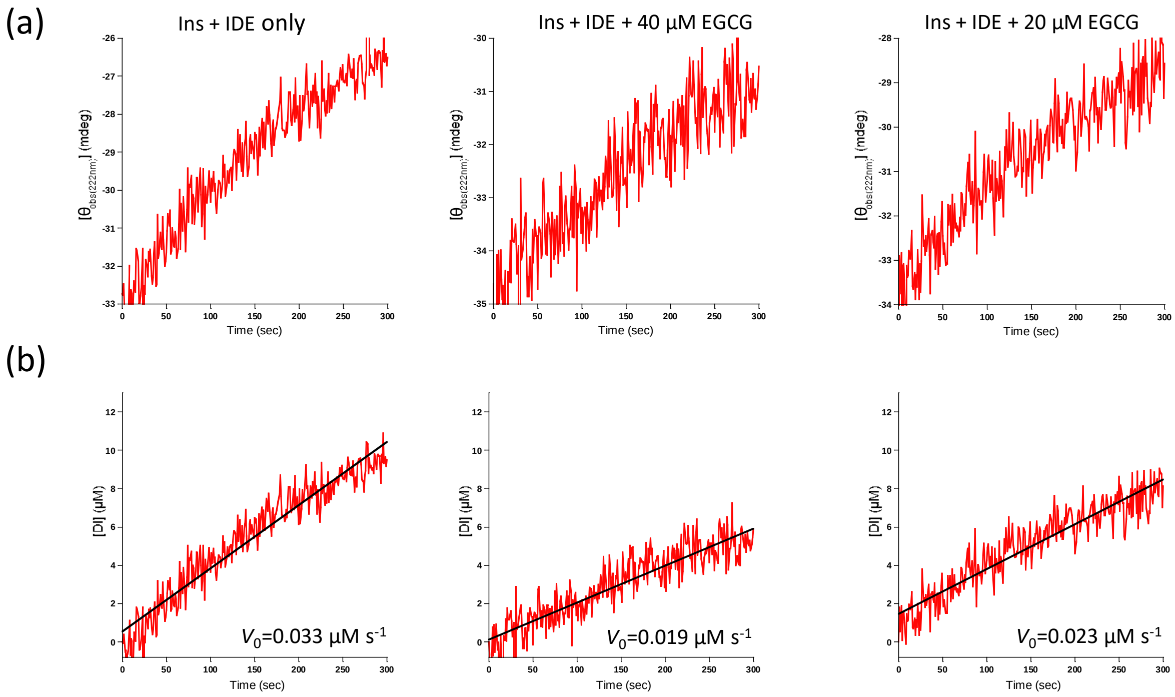

Figure S2. Early-stage kinetics of IDE-dependent degradation of insulin in the absence and presence of EGCG using observed ellipticity at 222 nm [$\theta_{obs(222\ nm)}$]. (a) Representative real-time plots of [$\theta_{obs(222\ nm)}$] versus initial digestion time (0 - 300 s) in the absence and presence of 40 and 20 μM EGCG at 37 ˚C. (b) Corresponding real-time plots of digested insulin [DI], calculated using Equation 1, versus digestion time. The R^2 values of the fitting of the data to straight lines were determined to be 0.92, 0.80, and 0.86 (from left to right). V_0 was obtained from the slope of each line. The concentration of insulin in these samples was set at 50 μM.

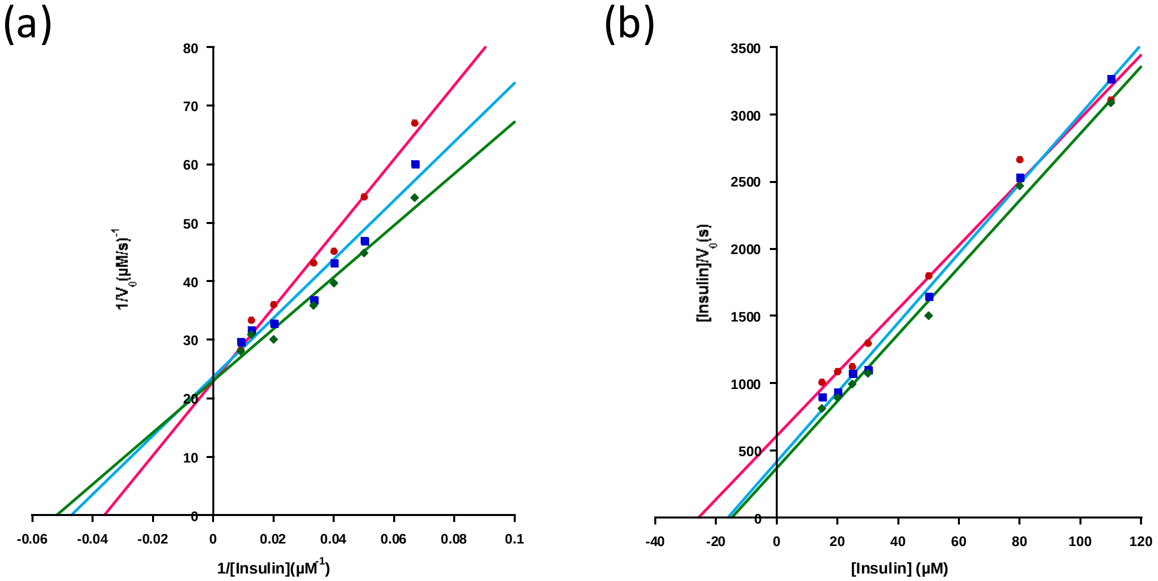

Figure S3. Kinetics of IDE-dependent degradation of insulin in the absence and presence of resveratrol at 37 °C. (a) Lineweaver-Burk and (b) Hanes-Woolf plots for insulin digests in the absence and presence of resveratrol are similar to one another indicating that the polyphenol has no effect on IDE's activity towards insulin. Plots for polyphenol concentrations of 0, 20 and 40 μM are shown in green, red and blue, respectively. Each data point represents the mean from three trials. The solid lines in (a) and (b) are fits to the Lineweaver-Burk and Hanes-Woolf equations, respectively.

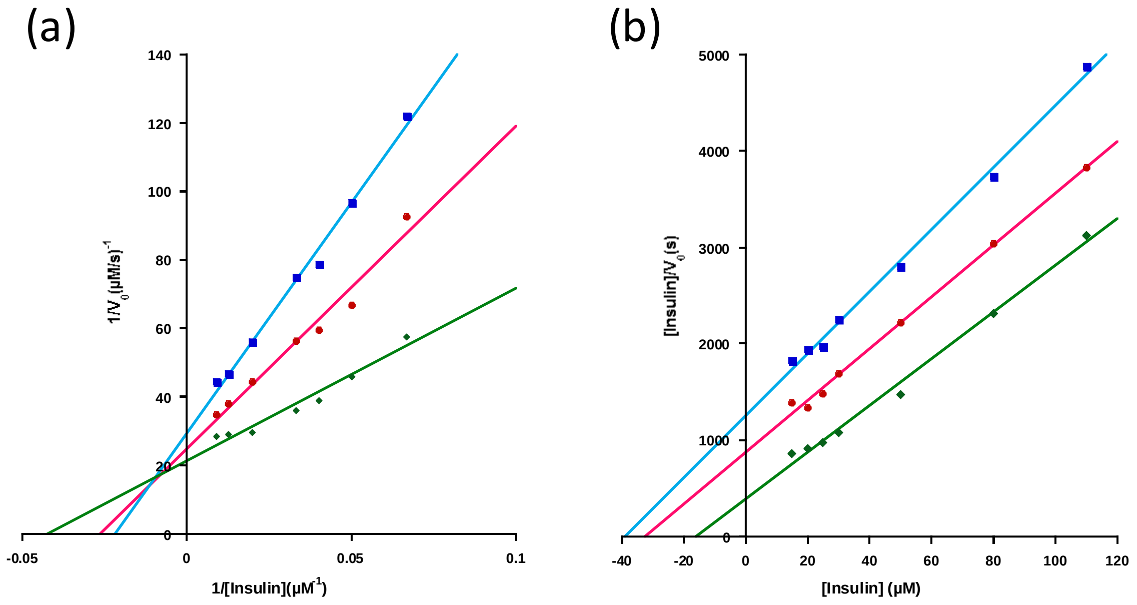

Figure S4. Kinetics of IDE-dependent degradation of insulin in the absence and presence of EGCG at 37 °C. (a) Lineweaver-Burk and (b) Hanes-Woolf plots for insulin digests in the absence and presence of EGCG show that the polyphenol inhibits IDE's activity towards insulin. Plots for polyphenol concentrations of 0, 20 and 40 μM are shown in green, red and blue, respectively. Each data point represents the mean from three trials. The solid lines in (a) and (b) are fits to the Lineweaver-Burk and Hanes-Woolf equations, respectively.

Table S1. Steady-state kinetic parameters of the IDE-dependent proteolysis of insulin determined from Lineweaver-Burk plots.

Polyphenol	K_M (M)	k_{cat} (s^{-1})	k_{cat}/K_M($M^{-1}S^{-1}$)
None	$2.4 \pm 0.2 \times 10^{-5}$	0.047 ± 0.0008	$2.0 \pm 0.2 \times 10^{3}$
None/Ethanol*	$2.1 \pm 0.2 \times 10^{-5}$	0.042 ± 0.001	$2.3 \pm 0.2 \times 10^{3}$
Resveratrol (20 μM)	$2.8 \pm 0.1 \times 10^{-5}$	0.044 ± 0.003	$1.7 \pm 0.4 \times 10^{3}$
Resveratrol (40 μM)	$1.9 \pm 0.1 \times 10^{-5}$	0.043 ± 0.003	$2.3 \pm 0.09\times 10^{3}$
EGCG (20 μM)	$4.3 \pm 3.3 \times 10^{-5}$	0.042 ± 0.001	$1.2 \pm 0.5 \times 10^{3}$
EGCG (40 μM)	$4.5 \pm 1.2 \times 10^{-5}$	0.034 ± 0.003	$0.74 \pm 0.2 \times 10^{3}$

*Control experiments to test the effect of ethanol that was used to dissolve resveratrol.

Table S2. Steady-state kinetic parameters of IDE-dependent proteolysis of insulin determined from Hanes-Woolf plots.

Polyphenol	K_M (M)	k_{cat} (s^{-1})	k_{cat}/K_M($M^{-1}S^{-1}$)
None	$1.6 \pm 0.3 \times 10^{-5}$	0.041 ± 0.0007	$2.5 \pm 0.5 \times 10^{3}$
None/Ethanol*	$1.5 \pm 0.3 \times 10^{-5}$	0.040 ± 0.001	$2.7 \pm 0.4 \times 10^{3}$
Resveratrol (20 μM)	$2.7 \pm 1.3 \times 10^{-5}$	0.043 ± 0.003	$1.8 \pm 0.7 \times 10^{3}$
Resveratrol (40 μM)	$1.6 \pm 0.3 \times 10^{-5}$	0.039 ± 0.004	$2.4 \pm 0.2\times 10^{3}$
EGCG (20 μM)	$3.3 \pm 1.4 \times 10^{-5}$	0.038 ± 0.003	$1.2 \pm 0.4 \times 10^{3}$
EGCG (40 μM)	$3.9 \pm 0.8 \times 10^{-5}$	0.031 ± 0.001	$0.80 \pm 0.2 \times 10^{3}$

*Control experiments to test the effect of ethanol that was used to dissolve resveratrol.
